# From penicillin to precision: reframing AMR in dentistry through time – past lessons, present risks, and future solutions

**DOI:** 10.1038/s41415-026-0173-y

**Published:** 2026-11-13

**Authors:** Lindsey Ann Edwards, Julie Ann Edwards, Yvonne Dailey

**Affiliations:** 387084393659072667776 Centre Host Microbiome Interactions, Faculty of Dentistry and Oral Craniofacial Sciences, Guy ´s Hospital, Guy ´s Tower, King ´s College London, London, United Kingdom; 299896065373163064393 King ´s College Hospital, Dental Institute, London, United Kingdom; 797924184228073532742 NHS England, North West, United Kingdom

## Abstract

Antimicrobial resistance (AMR) is a public health global threat projected to cause 10 million deaths annually by 2050 and a major health protection issue. Dentistry contributes approximately 10% of antibiotic prescriptions worldwide, often for conditions where antibiotics offer limited benefit. This article reframes AMR in dentistry through a historical, current, and future lens, integrating risk assessment principles, empirical prescribing trends, and precision strategies. We trace the trajectory from the penicillin era to modern stewardship challenges, examine the dental AMR burden and prescribing patterns using English Surveillance Programme for Antimicrobial Utilisation and Resistance 2023–2024 data, and explore innovative solutions including rapid diagnostics, microbiome restoration, and non-antibiotic therapies. Drawing on evolutionary insights from ancient microbiomes, we argue that AMR is not merely a clinical phenomenon, but a microbial ecological imbalance driven by industrialisation and antibiotic overuse. Future health protection strategies involving interdisciplinary working must combine stewardship with precision dentistry and microbial ecosystem restoration in a one-world setting to mitigate AMR risk while preserving microbial and host resilience. Public health plays an important role working with a range of other stakeholders/agencies in a multidisciplinary approach.

## Key points

- Antimicrobial resistance (AMR) in dentistry reflects disruption of the oral microbiome and microbial ecology, highlighting the need to view oral health, systemic health, and antibiotic stewardship as interconnected challenges.
- Delayed access to definitive dental care drives unnecessary antibiotic prescribing, while stewardship, prevention, and evidence-based operative treatment remain the most effective current AMR interventions.
- Dentists can help reduce AMR by prioritising definitive treatment over antibiotics where appropriate, promoting oral health and disease prevention to reduce the need for antibiotic prescribing, and preparing for future precision approaches including rapid diagnostics and microbiome-informed therapies.

## Antimicrobial resistance and the oral microbiome

Oral diseases represent one of the most prevalent non-communicable global health challenges, affecting 3.5–3.7 billion people – nearly 45% of the world's population. Untreated dental caries alone impacts approximately 2.5 billion individuals, making it the most common health condition worldwide. Severe periodontitis affects around 1 billion people, while complete tooth loss (edentulism) impacts 350 million, and oral cancer accounts for 380,000 new cases annually. This global picture is replicated in the recently published English Adult Oral Health Survey 2023,^1^ describing a 13-percentage-point increase in prevalence (among dentate adults) of having a combination of obvious primary and secondary coronal decay (28% [2009] – 41% [2023]). Further, 19% had one or more potentially urgent conditions, increasing to 31% of adults residing in the most deprived areas. These conditions are not confined to the mouth; they share risk factors with systemic diseases and influence overall health, immunity, and susceptibility to infection.^2^^,^^3^^,^^4^ Despite this enormous burden, oral health is often overlooked in global health strategies. Yet, the oral cavity hosts the second-most-diverse microbial community in the human body, comprising over 700 bacterial species.^5^ This microbiome plays a critical role in immune priming and maintaining mucosal homeostasis.^6^ The mouth serves as an entry point to the respiratory and digestive systems and is highly vascularised, with potential implications for the oral microbiome in other systemic diseases.^7^^,^^8^ Disruption, whether through disease or antibiotic exposure, can trigger systemic inflammation and increase vulnerability to chronic conditions such as diabetes, cardiovascular disease, and respiratory infections.^9^^,^^10^

The World Health Organization has declared a significant growing global threat of antimicrobial resistance (AMR).^11^ AMR is responsible for one in eight global deaths annually and is projected to cause 10 million deaths per year by 2050.^11^ Archaeological evidence indicates that antimicrobial resistance genes (ARGs) have existed for millennia. Still, their abundance has surged since the widespread use of antibiotics in the modern era.^12^^,^^13^^,^^14^^,^^15^ Dentistry contributes significantly to this problem: in the UK, dental practitioners account for approximately 10% of all antibiotic prescriptions in primary care, often for conditions where antibiotics offer limited benefit.^16^^,^^17^

Could you die from a ‘simple' dental infection? Sadly, yes, and AMR means this could become an everyday reality in all countries, regardless of income status. Bacteria and other microorganisms are evolving to resist the drugs traditionally used to kill them, such as antibiotics, transforming treatable infections into potentially deadly threats. AMR is often dubbed the ‘silent coming plague'^18^ jeopardising our ability to treat patients safely, crippling healthcare systems, and complicating care delivery for the most vulnerable in society. This critical global health challenge threatens to undo decades of medical progress and endanger millions of lives annually. If not addressed, AMR will suck trillions of pounds from the global economy^19^ and put pressure on already struggling healthcare systems.

Data from the Global Research on Antimicrobial Resistance project reveals the staggering toll of common bacterial infections.^11^ Young infants, the older population, and people with chronic illnesses (currently responsible for 41 million global deaths per year), or those undergoing cancer treatments or requiring surgical procedures are at the highest risk of infections. Infections disproportionately affect low- and middle-income countries.^20^ AMR is increasingly influenced by global environmental change; rising temperatures and ecosystem disruption are associated with enhanced bacterial proliferation, increased rates of horizontal gene transfer, and wider dissemination of resistance determinants across environmental and clinical reservoirs.^21^^,^^22^^,^^23^ AMR is closely linked to food security, particularly through its impact on agricultural systems and the sustainability of livestock production,^24^ thus underscoring the urgent need for targeted interventions and strengthened health systems, highlighting the utmost urgency of addressing this escalating global threat.^25^

The majority of antimicrobial prescribing in dentistry globally is associated with the management of acute dental infections (acute apical abscess, irreversible pulpitis, pericoronitis) often where operative treatment is delayed or inaccessible.^17^ However, antibiotic exposure extends across broader clinical scenarios, including prophylactic use such as for prevention of infective endocarditis for those at risk, and post-operative prescribing. Untreated caries may progress to infection, with antibiotics sometimes used in place of definitive care, reflecting underlying issues of access and oral health inequalities. Systemic antibiotics are not routinely indicated for periodontitis and should only be used as adjunctive therapy in selected cases following appropriate staging and grading, in accordance with the 2017 World Workshop classification^26^ and established clinical guidelines.^27^ Such use in indicated patient cases would usually be undertaken by a specialist, and the combination of choice in the UK involves amoxicillin and metronidazole **–** a broad-spectrum combination effective against anaerobic and Gram-negative pathogens such as *Porphyromonas gingivalis*, *Aggregatibacter actinomycetemcomitans*, and *Tannerella forsythia*. However, it is also important to note that such regimens disrupt both oral and gut microbiomes, potentially promoting AMR and cross-resistance to other antibiotic classes. Beyond clinical settings, antibiotic residues entering wastewater systems further propagate resistance and environmental degradation.^13^^,^^21^^,^^22^^,^^23^^,^^24^ This highlights that AMR in dentistry is driven more by prescribing behaviours and system-level factors than by specific diagnoses alone. In dentistry, adherence to existing diagnostic frameworks and treatment guidelines offers immediate opportunities to reduce unnecessary antibiotic use without requiring new technologies or therapeutic innovations.

Delayed or inadequate intervention in dental infections can lead to rapid progression, sepsis, airway obstruction, and even death, underscoring the critical need for timely access to definitive care. Where access is limited, antibiotics are often prescribed as a preventive measure – highlighting the tension between immediate patient safety and long-term AMR risk. Improved oral hygiene and maintenance of a balanced oral microbiome may contribute to reduced systemic inflammatory burden and support overall health.^28^

**Fig. 1:**
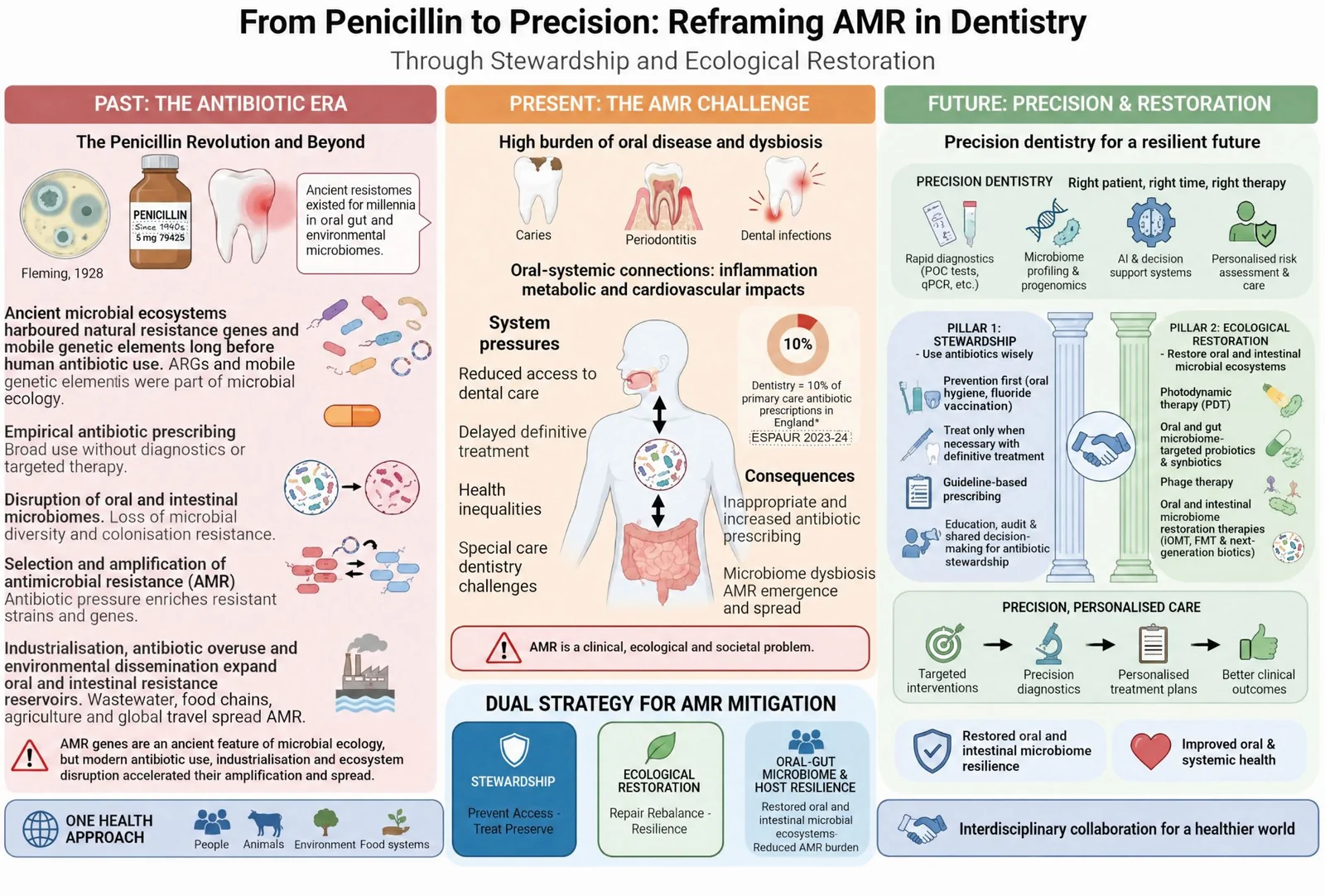
From penicillin to precision: the evolution of AMR in dentistry. Conceptual framework illustrating the transition from empirical antibiotic use in dentistry (past/present), to the present where antimicrobial resistance poses a major challenge, and finally to the future. Emphasising precision, microbiome-informed care. Historical antibiotic exposure and industrialisation have altered microbial ecology and amplified antimicrobial resistance. Future mitigation depends on combining antimicrobial stewardship, prevention and equitable access with rapid diagnostics, precision therapeutics, and ecological restoration of the oral microbiome within a One Health Framework. Figure created using BioRender.com

This is an area in which multidisciplinary collaboration is vitally important, including public health dentistry, both directly in relation to population health and indirectly through health promotion and health systems. The following sections will look at AMR learning lessons from the past, present risks, and future solutions starting with learning from the past. The evolution of antimicrobial use in dentistry, the emergence of AMR, and future precision-based interventions are summarised in Figure 1.

## Past lessons: antibiotics, ecology, and the oral microbiome

Antibiotics revolutionised dentistry in the mid-20^th^ century, with the discovery of penicillin in 1928 and its mass production in the 1940s. Clinicians gained the ability to manage odontogenic infections and undertake surgical prophylaxis with reduced morbidity.^17^ However, early prescribing practices lacked stewardship principles with widespread empirical prescribing, often for conditions such as irreversible pulpitis, minor periodontal disease or for pain relief in the absence of infection, a practice now considered inappropriate.^29^^,^^30^ This empirical legacy has set the stage for antimicrobial resistance emergence.^13^ Dentistry today accounts for approximately 10–15% of outpatient antibiotic prescriptions globally, with amoxicillin and metronidazole dominating usage patterns.^31^ This has driven resistance among oral microorganisms, including pathogens such as *Streptococcus mutans* and anaerobic species, particularly in biofilm-associated infections where the structured microbial community, extracellular matrix, and reduced metabolic activity limit antibiotic penetration and efficacy.^32^^,^^33^

While the antibiotic era marked a turning point, the foundations of antimicrobial resistance predate modern medicine. Over 1.5 billion years of co-evolution, humans and their microbiota have developed critical interdependent ecosystems, that regulate immunity, metabolism, and pathogen defence. The microbiome represents both a reservoir of beneficial functions and a potential source of AMR. It is a highly diverse community that plays a central role in maintaining mucosal homeostasis.^34^ Within this ecological framework, ARGs are not merely products of contemporary antibiotic use, but components of a broader resistome shaped by long-term evolutionary pressures.

Many individuals carry ARGs, yet most do not develop resistant infections, as gene carriage alone is insufficient to drive pathology or treatment failure. ARGs therefore cannot be understood solely as clinical threats; they are longstanding components of microbial communities and may contribute to microbial competition, stability, and ecological balance. As part of the broader microbial pangenome, resistance mechanisms have historically enabled coexistence between hosts and their microbiota.^35^^,^^36^ The key challenge is not the eradication of ARGs, but identifying which variants are mobilised, transferred, and expressed in ways that lead to clinically significant infection. In this sense, antimicrobial resistance represents a ‘wicked' problem – one that cannot be eliminated, but must be actively managed through improved understanding, surveillance, and stewardship across interconnected biological and societal systems.^37^

Advances in paleogenomics and high throughput biomolecular sequencing have enabled detailed reconstruction of ancient microbiomes, including those preserved in dental calculus. Palaeofaeces and dental calculus show that pre-industrial gut and oral microbiomes cluster with those of non-industrialised modern populations, and are distinct from industrialised profiles.^35^^,^^38^^,^^39^^,^^40^^,^^41^^,^^42^^,^^43^^,^^44^ Importantly, these ancient samples exhibit:

- Lower abundance of antibiotic resistance genes which are genes that enable bacteria to survive antibiotic exposure, and mucin-degrading genes, which are linked to disruption of the normal balance of intestinal microorganisms (known as intestinal dysbiosis) and inflammatory diseases today.^43^^,^^44^^,^^45^ Reduced mucin-degrading genes, reflect limited reliance on host-derived glycans in pre-industrial microbial ecosystems, suggesting pre-industrial microbiomes were more diverse and stable with access to high fibre diets, so less need to degrade mucin
- Enrichment of mobile genetic elements (MGEs) which are pieces of deoxyribonucleic acid that can move between bacteria and transfer traits such as antibiotic resistance, indicating dynamic but balanced microbial gene exchange that supported adaptability and resilience in past populations.

Rather than viewing AMR solely as pathogen resistance, these findings support an ecological perspective in which clinically significant resistance emerges when ARGs are selected, amplified, and mobilised within permissive host–microbial environments. Human activities including industrialisation, international travel, antibiotic overuse, processed diets, and reduced microbial exposure, has disrupted the equilibrium, reshaping ARG profiles and MGE activity in ways that erode health. This reframes AMR as a symptom of microbial ecosystem disruption rather than just pathogen resistance.

In dentistry, these ecological dynamics are especially relevant. The oral cavity represents a biofilm-dominated environment in which microbial composition is highly responsive to diet, hygiene practices, and antimicrobial exposure. Oral dysbiosis can therefore drive both local disease and broader systemic effects. Oral diseases such as caries and periodontitis have multiple risk factors and are not caused by single pathogens but involve shifts in microbial community structure and function. In this context, the use of antibiotics for their management targets only a subset of organisms and may further destabilise microbial ecosystems, thus, limiting their long-term effectiveness and contributing to resistance selection.

Modern investigations of the human microbiome have demonstrated its central role in health and chronic disease, raising important questions about how microbial ecology, diversity, and function have changed over time.^46^^,^^47^^,^^48^ Increasingly, microbiomes are recognised as modifiers of host traits, including immunity, metabolism, reproduction, and behaviour. This systems-level perspective is reflected in the concept of the ‘holobiont', in which the host and its associated microbiota function as an integrated biological unit. Holo-omics approaches extend this framework by integrating host and microbiome data (microbial genomics, transcriptomics, metabolomics) to examine how these interactions influence metabolic cooperation, immune modulation, and trait inheritance.^47^^,^^48^ It is important to distinguish industrialisation from the antibiotic era. Industrialisation, spanning the past two to three centuries, introduced profound changes in human environments, including urbanisation, dietary shifts toward processed and sugar-rich foods, reduced microbial exposure, and altered patterns of disease. By contrast, the widespread use of antibiotics is a much more recent phenomenon, emerging only in the mid-20^th^ century. These overlapping but distinct transitions have jointly shaped the composition and function of the human microbiome. Driving species loss, reducing functional diversity, and increasing dysbiosis factors that underpin the emergence and spread of clinically relevant AMR and contributing to inflammatory, metabolic, and infectious diseases.^15^^,^^22^^,^^49^^,^^50^

What many people do not realise is that oral health is critical to overall systemic health. The oral cavity is a major immunological interface, hosting complex microbial communities that prime immune responses and maintain mucosal tolerance.^51^^,^^52^^,^^53^ Disruption of this balance through dysbiosis or untreated oral disease can amplify systemic inflammation, increasing susceptibility to chronic conditions such as diabetes, cardiovascular disease, and rheumatoid arthritis.^54^^,^^55^^,^^56^ Poor oral health also heightens vulnerability to infections and sepsis, particularly in older or immunocompromised individuals.^57^ This makes antimicrobial stewardship in dentistry not only a local issue but a global health imperative.^16^^,^^58^^,^^59^^,^^60^

## The AMR burden present risks: oral health and stewardship integration, and dental access

### Embedding oral health as integral to systemic health

Reducing AMR in dentistry requires oral health to be treated as integral to systemic health, with antimicrobial stewardship embedded in routine practice. This depends not only on clinical behaviour, but also on addressing structural and political barriers that prevent access to timely dental care across UK and global systems.

Public health messaging that emphasises oral health as a foundation for systemic immunity and chronic disease prevention could transform perceptions and behaviours. Emphasising prevention – including effective oral hygiene using a fluoride toothpaste, reduced free sugar intake, avoiding tobacco use and minimising alcohol and equitable access to dental care. Positioning oral care as a critical component of overall health – not merely cosmetic – can drive better oral-hygiene practices, reduce infection incidence and the need for antibiotic prescribing. Reflected in initiatives by organisations such as the World Health Organization; FDI World Dental Federation; British Dental Association (BDA); Oral Health Foundation UK; Centres for Disease Control and Prevention; and Health Resources and Services Administration.

Importantly, these campaigns should also highlight the role of a healthy microbiome, both oral and gut, in maintaining immune balance and metabolic health. A resilient microbiome supports mucosal immunity, reduces inflammation, and helps prevent opportunistic infections. Integrating microbiome awareness into oral health education can encourage lifestyle choices that preserve microbial diversity, such as balanced diets, reduced unnecessary antibiotic exposure, and avoidance of overly aggressive antimicrobial oral products.^51^^,^^52^^,^^53^^,^^61^^,^^62^ Embedding this message into national health campaigns and AMR strategies would strengthen prevention at the population level.^63^^,^^64^ AMR is now recognised as a critical health protection issue. The UK's 2024–2029 National Action Plan (NAP) aims to reduce antimicrobial use by 5% from the 2019 baseline and achieve 70% Access-category antibiotic use across healthcare.^65^ Dentistry must align with these goals while addressing unique ecological and behavioural drivers of resistance within oral health.

### Policy and stewardship integration

The UK 2024–2029 NAP on AMR explicitly calls for dentistry to align with national targets: reducing total antimicrobial use.^65^ The UK Health Security Agency Dental Antimicrobial Stewardship (AMS) Toolkit provides practical guidance,^66^ but uptake remains inconsistent, highlighting the need to align policy, training and service design in the UK and globally. The FDI World Dental Federation has actively raised awareness of AMS in dentistry.^58^^,^^67^ Additionally, it supports national dental associations in implementing AMS programmes to combat AMR.^17^^,^^65^^,^^66^^,^^68^ However, successful implementation of stewardship policies depends not only on professional and institutional commitment, but also on public engagement and support. Recent international deliberative polling led by Dame Sally Davies, UK Special Envoy on AMR demonstrated strong public support for AMR policy interventions aligned with the 2024 United Nations General Assembly Political Declaration on AMR across six middle-income countries. Importantly, the study also revealed substantial diversity in perspectives shaped by differing structural, economic, environmental and health system contexts, highlighting the need for stewardship programmes and national action plans to be adapted to local priorities while remaining aligned with global AMR objectives. These findings suggest that public engagement is critical for securing the societal mandate needed to translate international commitments into effective national policy and sustained antimicrobial stewardship practice.^69^^,^^70^

### Empirical prescribing

In the UK, amoxicillin, followed by metronidazole, remain the most prescribed agents. Amoxicillin has been the antimicrobial of choice for acute dental infections; however, in October 2020, the Scottish Dental Clinical Effectiveness Programme recommended phenoxymethylpenicillin as the first line antimicrobial for dental infections^71^^,^^72^ because of its narrower antimicrobial spectrum and lower potential to drive antimicrobial resistance. Prescribing practice has shifted towards greater use of phenoxymethylpenicillin. The NHS Business Services Authority dental prescribing surveillance data provide evidence of these changing prescribing patterns in England.^73^ The English Surveillance Programme for Antimicrobial Utilisation and Resistance (ESPAUR) report confirms that up to 80% of dental antibiotic prescriptions may be unnecessary, driven by patient pressure and diagnostic uncertainty.^60^

### Political and media dimensions

The UK Steele Report emphasises that increased primary care funding alone will not resolve dental access problems.^74^ Without wider system reform, short-term urgent appointment pledges are unlikely to address the underlying pressures that drive antibiotic use.^75^ The UK 2024 dental recovery plan is attempting to address the outdated contract currency in England, and incentives for providing prevention and complex care.^76^ The outcomes of this still remain to be seen.^77^^,^^78^^,^^79^ Moreover, media coverage has often focused heavily on access difficulties; potentially reinforcing public disengagement. There is a pivotal opportunity for the media to broaden and rebalance the narrative to include oral health, microbiome resilience and increasing awareness of AMR, which could improve public understanding, engagement, and support for reform.^80^^,^^81^

### Dental access

#### Urgent and unscheduled care

Limited access to unscheduled and urgent dental care may have a direct impact on antibiotic use. When definitive treatment such as extraction or drainage is delayed, antibiotics may be used as a substitute for treatment. During COVID-19, restricted access was associated with a marked increase in dental antibiotic prescribing across several developed countries, including: Canada, where the province of Alberta recorded a 76% surge; a 49% rise was seen in Scotland; and 25% in England.^82^ Although rates have since fallen, they remain above pre-pandemic levels across the UK, demonstrating how quickly stewardship is weakened when access collapses.^83^^,^^84^^,^^85^^,^^86^

In the UK, dentists have stressed that antibiotics do not cure toothache, yet patient expectations and remote consultations during the pandemic reinforced inappropriate narratives. The BDA and stewardship leaders argue that the most effective AMR protection is timely access to definitive operative care, rather than reliance on antibiotics.^85^^,^^86^

### AMR and special care dentistry in England

The ESPAUR report,^60^ highlighted that people living in the most deprived communities in England face up to 47% higher rates of resistant bacteria than those living in the least deprived areas. These findings mirror oral health data, where both vulnerable adults and children residing in deprived areas suffer the worst oral health.^87^ Special consideration should be given to people with existing vulnerabilities. In the UK, the specialty of special care dentistry (SCD) provides oral health care for some of the more vulnerable adult patients with physical, sensory, intellectual, mental, medical, emotional, or social impairments. These individuals often require complex interventions, sometimes under sedation or general anaesthesia (GA), and are disproportionately affected in accessing care in a timely manner. NHS England's clinical standards highlight that SCD is delivered across primary care, hospitals, and community settings, but workforce shortages and fragmented referral pathways limit timely treatment.^87^

### Why access matters for AMR in SCD

Patients in SCD frequently present with advanced oral disease due to delayed care, increasing the likelihood of systemic infection risk and hospital admissions. When definitive treatment (e.g., extractions under GA) is delayed, clinicians often feel under pressure to resort to prescribing antibiotics. This is particularly problematic because:

- Many SCD patients are immunocompromised or medically complex, making prescribers more cautious and prone to prophylactic or empirical antibiotic use
- Guidelines for urgent care in SCD emphasise that definitive operative treatment – not antibiotics – is the gold standard for managing acute dental conditions. However, national evidence reviews highlight that limited access to urgent operative care leads to inappropriate antibiotic prescribing, particularly for patients with additional needs, thus undermining this principle.^88^^,^^89^^,^^90^

### Current trends and risks

Evidence suggests that antibiotic prescribing spikes when urgent care slots are unavailable, a trend mirrored in SCD where treatment often depends on specialist facilities and anaesthesia support.^16^

During COVID-19, antibiotic use surged by 25% in dentistry overall; anecdotal reports suggest SCD patients were disproportionately affected due to suspension of GA lists and domiciliary visits.^91^

### Policy and stewardship implications for SCD

The UK's 2024–2029 National Action Plan on AMR calls for dentistry to reduce antimicrobial use and achieve 70% Access-category prescribing. For SCD, this means embedding AMS into care pathways and specialist training and ensuring urgent access slots for complex cases.^16^ The British Society for Special Care Dentistry advocates for integrated care pathways and domiciliary services to prevent infection escalation and reduce reliance on antibiotics.^92^ Contract reform and funding for GA lists, domiciliary care, and community clinics are essential to meet these goals. Without systemic changes, vulnerable patients will remain at high risk for inappropriate antibiotic exposure and AMR.

## Future solutions: from empirical to precision

Addressing AMR in dentistry depends firstly on the consistent application of already existing strategies. At present, the most effective interventions lie in evidence-based prevention, timely improved access to care, training and adherence to evidence-based prescribing in clinical practice. Public health messaging is central to this effort, clearly emphasising the link between oral health, systemic health and AMR. Alongside these foundational approaches, periodontal therapy is gradually moving from empirical prescribing towards precision-based, individualised care, supported by stewardship frameworks, diagnostic innovation, non-antibiotic alternatives and ecological insights from ancient microbiomes.

### What can be done now: prevention, access, and stewardship

Prevention is vitally important. The most impactful strategies for mitigating AMR will come from prevention as reducing the burden of oral disease directly decreases the need for antibiotic prescribing. Interventions such as effective oral hygiene with an age-appropriate fluoride toothpaste, water fluoridation, dietary modification – particularly reduced free sugar intake and diets that support microbial balance,^61^^,^^62^ – and early disease management of dental disease are well established but remain underutilised. These measures not only improve oral health outcomes but also reduce infection incidence and therefore, consequently, antibiotic exposure.

Timely access to definitive dental care is equally important and a critical determinant of antibiotic use. Where operative treatment – such as drainage or extraction – is delayed or unavailable, antibiotics are more likely to be prescribed as a substitute. Addressing inequalities in access, particularly for vulnerable groups populations and those requiring specialist care, is therefore core/central to AMR intervention mitigation. Ensuring availability of urgent dental care and definitive treatment pathways represents one of the most immediate and effective system-level interventions.

Effective communication and education of society are essential. Patient expectations regarding antibiotics for dental pain continue to drive prescribing pressures, while variation in clinician adherence to guidelines contributes to unnecessary antibiotic use. Public health messaging on dental pain, that emphasises the link between oral health, systemic health and AMR can support behaviour change at the population level. For clinicians, reinforcement of guideline-based prescribing can reduce unnecessary antibiotic use. Contemporary clinical decision making – including appropriate staging and grading of periodontal disease and avoidance of antibiotics in the absence of systemic involvement – is essential.

Dental antimicrobial stewardship should prioritise definitive treatment over use of antibiotics. This remains the cornerstone of effective practice. In dentistry, this primarily involves prioritising operative treatment and avoiding empirical prescribing where possible, reserving antibiotics for clearly indicated cases. Thus importantly, the most effective current stewardship interventions do not require new technologies but depend on consistent application of existing clinical frameworks.^67^

In the UK NAP sets ambitious targets to reduce overall antimicrobial use and curb reliance on watch and reserve category antibiotics.^65^ Dentistry, which accounts for around one in ten primary care antibiotic prescriptions,^16^ is a key focus. Practical frameworks such as the TARGET Dental Antimicrobial Stewardship Toolkit, developed by the UK Health Security Agency ESPAUR subgroup, provide clinicians with educational resources and prescribing guidance.^66^ At the global level, the FDI World Dental Federation has adopted stewardship policies urging dentists to integrate responsible prescribing into national action plans.^67^ Despite these resources, uptake remains uneven, with surveys showing low awareness among dentists, but high willingness to adopt change if supported by training and system-level incentives. Capitalising on this willingness represents a significant opportunity. Investment in continuing professional development, targeted education on oral-systemic health, and practical stewardship training can empower clinicians to reduce unnecessary antibiotic use. Professional organisations and journals, including the *British Dental Journal*, play a critical role in advocating for structural changes – such as incentivising preventive care, improving access to timely treatment, and embedding stewardship within contractual frameworks. Aligning education, incentives, and clinical practice is therefore essential for sustained reductions in antimicrobial use.

### Improving practice: moving beyond empirical prescribing

Alongside stewardship, precision dentistry is emerging as a transformative paradigm.^93^ Rapid diagnostics such as real-time quantitative polymerase chain reaction panels and metagenomic sequencing can identify pathogenic signatures and resistance genes in real-time,^13^ while artificial intelligence (AI)-driven decision support systems trained on large datasets can predict disease progression and stratify patients by risk. Linking diagnostic outputs with prescribing databases creates feedback loops that monitor antibiotic use and outcomes, embedding stewardship into daily practice. Pilot studies already suggest that combining rapid diagnostics with AI can reduce unnecessary antibiotic prescriptions by nearly a third, without compromising efficacy.^94^^,^^95^

### Microbiome modulation and non-antibiotic alternative treatments

The move toward precision care also opens the door to non-antibiotic alternatives. Photodynamic therapy has been shown in systematic reviews to reduce periodontal pathogens and improve clinical outcomes when used adjunctively.^96^^,^^97^ Probiotics and synbiotics, which combine beneficial microorganisms with nutrients that enhance their survival and colonisation, are gaining traction, with umbrella meta-analyses demonstrating reductions in probing depth, bleeding on probing, and plaque indices.^98^^,^^99^

Inspired by macroecology, microbiome engineering now applies niche dynamics and community assembly rules to design stable, functional microbial consortia.^100^ This is transforming medicine, agriculture, and conservation by enabling precision modulation of the microbiome. Bacteriophage therapy is another promising avenue: systematic reviews highlight its potential to target resistant biofilms in oral infections, including periodontitis.^101^^,^^102^ Other innovations, antimicrobial peptides, quorum-sensing inhibitors, and nanomaterials designed to disrupt biofilm architecture, illustrate the breadth of alternatives poised to complement or replace conventional antibiotics.^103^^,^^104^^,^^105^ Underlying all of these developments is a recognition of the co-evolutionary relationship between humans and their microbiota. Archaeogenomic studies of ancient oral microbiomes reveal lower abundance of ARGs and greater functional diversity compared to modern dysbiotic communities.^106^^,^^107^^,^^108^ This suggests that industrial-era antibiotic use has narrowed microbial diversity and expanded resistance reservoirs. Restoration strategies must therefore aim not only to suppress pathogens but to rebuild ecological resilience.

One emerging approach is oral microbiota transfer (OMT), modelled on the success of faecal microbiota transplantation in gastrointestinal medicine.^109^^,^^110^ Early pilot studies suggest that transferring a balanced oral microbial community from healthy donors could help re-establish ecological stability in patients with severe oral dysbiosis. While still experimental, OMT represents a radical but potentially transformative intervention, offering a way to restore microbial diversity and function rather than simply suppress pathogens. Combined with dietary interventions rich in polyphenols and prebiotics, and stewardship practices informed by pre-industrial baselines, microbiota transfer may become a cornerstone of future periodontal care.

Taken together, these strands – policy-driven stewardship, precision diagnostics, non-antibiotic alternatives, and microbiome restoration through strategies such as microbiota transfer – point toward a hybrid model of periodontal therapy. Population-level evidence will continue to underpin guidelines, but individualised diagnostics and ecological insights will increasingly shape treatment decisions, working closely with patients and the public. By moving beyond empirical prescribing toward predictive, personalised interventions, dentistry can improve patient outcomes, safeguard the microbiome, and contribute meaningfully to the global effort against antimicrobial resistance.

### AMR as a ‘wicked problem': integrating systems-level solutions

AMR is increasingly recognised as a ‘wicked' problem – characterised by complex, interdependent drivers spanning biological, clinical, environmental, and societal systems.^37^ It cannot be eradicated but must be continuously managed. This perspective reframes AMR in dentistry: not simply as a consequence of antibiotic use, but as a manifestation of ecological disruption within the oral microbiome, influenced by healthcare access, behaviour, and wider environmental change. Technological innovation will play an important role in improving diagnostics and enabling more targeted interventions. However, sustainable progress will depend on integrating these advances with foundational strategies *–* prevention, access to care, education, and stewardship – rather than relying on any single solution.

## Conclusions

Dentistry stands at a crossroads from the public health empirical legacy of penicillin to the promise of precision. This paper argues that AMR mitigation requires a dual strategy – robust stewardship and ecological restoration – underpinned by One Health governance, surveillance, and risk assessment approaches and informed by evolutionary insights. The COVID-19 pandemic exposed vulnerabilities in dental prescribing and the need for resilient, data-driven systems. Rapid diagnostics, personalised prescribing and microbiome-based therapies offer future potential, but the most immediate and impactful gains for now will come from system-wide collaboration underpinned by public health principles, to strengthen and embed prevention, equitable access to timely care, and global wide adherence to evidence based practice and education. By moving beyond empirical prescribing towards more informed, system-integrated, informed care approaches, dentistry has a unique opportunity to contribute meaningfully to addressing AMR while preserving microbial and host resilience.
